# Using venous blood gas analysis in the assessment of COPD exacerbations: a prospective cohort study

**DOI:** 10.1136/thoraxjnl-2015-207573

**Published:** 2015-12-01

**Authors:** Tricia M McKeever, Glenn Hearson, Gemma Housley, Catherine Reynolds, William Kinnear, Tim W Harrison, Anne-Maree Kelly, Dominick E Shaw

**Affiliations:** 1 Division of Epidemiology, University of Nottingham, Nottingham, UK; 2 Respiratory Research Unit, Division of Respiratory Medicine, University of Nottingham, Nottingham, UK; 3 Medical Informatics, East Midlands Academic Health Sciences Network, Nottingham, UK; 4 Respiratory Medicine, Nottingham University Hospital Trust, Nottingham, UK; 5 Emergency Medicine, Joseph Epstein Centre for Emergency Medicine Research, Western Health, St Albans, Victoria, Australia

**Keywords:** COPD Exacerbations

## Abstract

**Introduction:**

Identifying acute hypercapnic respiratory failure is crucial in the initial management of acute exacerbations of COPD. Guidelines recommend obtaining arterial blood samples but these are more difficult to obtain than venous. We assessed whether blood gas values derived from venous blood could replace arterial at initial assessment.

**Methods:**

Patients requiring hospital treatment for an exacerbation of COPD had paired arterial and venous samples taken. Bland–Altman analyses were performed to assess agreement between arterial and venous pH, CO_2_ and 
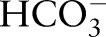
. The relationship between SpO_2_ and SaO_2_ was assessed. The number of attempts and pain scores for each sample were measured.

**Results:**

234 patients were studied. There was good agreement between arterial and venous measures of pH and 
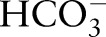
 (mean difference 0.03 and −0.04, limits of agreement −0.05 to 0.11 and −2.90 to 2.82, respectively), and between SaO_2_ and SpO_2_ (in patients with an SpO_2_ of >80%). Arterial sampling required more attempts and was more painful than venous (mean pain score 4 (IQR 2–5) and 1 (IQR 0–2), respectively, p<0.001).

**Conclusions:**

Arterial sampling is more difficult and more painful than venous sampling. There is good agreement between pH and 
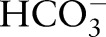
 values derived from venous and arterial blood, and between pulse oximetry and arterial blood gas oxygen saturations. These agreements could allow the initial assessment of COPD exacerbations to be based on venous blood gas analysis and pulse oximetry, simplifying the care pathway and improving the patient experience.

**Key messages**

**What is the key question?**

- Can venous blood gas analysis replace arterial blood gas sampling in the initial assessment of patients with COPD exacerbations?

**What is the bottom line?**

- Over two-thirds of arterial blood gas samples could be replaced by the simpler and safer use of venous blood gas analysis.

**Why read on?**

- This paper describes agreement between arteriovenous measures for key blood gas parameters and presents a simple algorithm for the substitution of arterial blood gas sampling with venous in the initial management of patients admitted to hospital with a COPD exacerbation.

## Introduction

Exacerbations of COPD are the second most common cause of emergency hospital admission in the UK, with an estimated 94 000 per year.^1^ COPD exacerbations have a very high risk of mortality; 50% of people with a severe exacerbation will die within 4 years of an admission.^1^

The recognition that high flow oxygen therapy can induce hypercapnia in susceptible patients during exacerbations of COPD,^2^ and that respiratory acidosis is associated with a worse outcome^3^ ^4^ led to a rise in arterial blood gas (ABG) sampling to measure pH, PaCO_2_, PaO_2_ and 
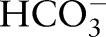
. The current National Institute for Health and Care Excellence (NICE) COPD guidelines recommend obtaining an ABG in all patients admitted to hospital with a COPD exacerbation.^5^ Arterial sampling is more technically difficult and reportedly more painful^6^ than venous blood gas (VBG) sampling. Administration of local anaesthetic prior to arterial sampling is recommended but seldom used, as shown in a recent survey of junior doctors where 91% never or rarely used local anaesthesia when performing arterial puncture.^7^ Using less invasive measures of pCO_2_ and SaO_2_ could greatly benefit patients by both decreasing pain and streamlining the care pathway.

Recent meta-analysis data suggest good agreement between venous and arterial measurements of pH, 
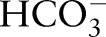
 and base excess.^8–11^ In diabetes care arterial sampling has been replaced with venous for the monitoring of diabetic ketoacidosis.^12^ The use of venous samples to guide treatment in COPD exacerbations has been limited, perhaps because the relationship between arterial and venous measures of CO_2_ is less strong, although a PvCO_2_ of >6 kPa has been shown to have 100% (95% CI 97% to 100%) sensitivity in identifying patients with clinically relevant hypercapnia.^13^

We set out to assess the relationship between arterial and venous measures of PCO_2_, pH and 
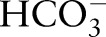
, and between arterial and pulse oximetry oxygen saturations during exacerbations of COPD, in order to establish whether VBG analysis combined with pulse oximetry could replace ABG analysis in the initial assessment of COPD exacerbations.

## Methods

Patients admitted to Nottingham University Hospitals Trust with a doctor-diagnosed exacerbation of COPD were considered for inclusion. Patients were included in the study unless they refused. If they were unable to give informed consent an approved alternative decision maker was approached. Recruited patients had an ABG and pulse oximetry performed by a junior doctor as per routine care, and a parallel paired VBG sample. Care was guided by the ABG results as per current clinical guidelines.

Arterial samples were collected via a heparinised needle and syringe system, and venous samples were aspirated into a separate heparinised blood gas syringe via a butterfly needle or needle. Samples were processed as soon as possible on the same ward-based blood gas analyser. Analysers were calibrated regularly in accordance with the department of medical physics standard operating procedure. Research nurses collected data on demographics, body mass index, smoking status, pain scores (visual analogue scores 0=no pain, 10=worst pain imaginable) for arterial and venous sampling and the number of attempts taken to acquire each sample.

The main outcomes were the agreements between ABG and VBG parameters, and between ABG and pulse oximetry measures of oxygen saturation. Secondary outcome measures included pain scores, and the number of attempts taken to obtain blood.

### Statistical analysis

Agreements between venous and arterial samples for CO_2_, pH and 
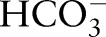
, and between SaO_2_ and SpO_2_ were assessed using the Bland–Altman method. Previous studies found 40% of patients attending the Emergency Department with an exacerbation of COPD had arterial hypercarbia, and it was estimated that 200 patients would allow us to calculate the sensitivity of the venous CO_2_ screening threshold for detection of arterial hypercarbia with CIs of <5%.^9^ ^13^ Using this sample size of 200, our 95% CI around the limits of agreement are estimated at 0.24× the SD of the mean difference for which pH is ±0.001.^14^

Missing data were not imputed. Receiver operating characteristic curves were used to estimate how venous CO_2_, pH, 
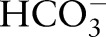
 predicted arterial values and how SpO_2_ predicted SaO_2_. Area under the curve, sensitivity and specificity were calculated to predict an arterial pH ≥7.35 and bicarbonate ≥21, and for the pulse oximetry result to predict an SaO_2_ of ≥92%.

## Results

Over the time course of the study (from 3 February 2013 to 10 January 2014), there were 1376 admissions with a coded diagnosis of COPD exacerbation. Of these, 234 participants were recruited and had at least one paired sample of blood gases. Twelve patients declined study participation. The mean age of the population was 71 years (SD 10.8) and 50% of the population was male. Characteristics of the population are shown in table 1.

**Table 1:** Characteristics of study population

	n	N
Age (years) mean (SD)	71.0 (10.8)	234
Sex N (%)		
Male	118 (50.4)	
Female	116 (49.6)	
BMI (kg/m^2^) mean (SD)	26.2 (7.9)	223
Smoking status N (%)		230
Never	14 (6.1)	
Ex	146 (63.5)	
Current	70 (30.4)	
Clinical measures mean (SD)		
Heart rate (bpm)	98.7 (20.8)	234
Respiratory rate	23.6 (5.9)	234
Systolic blood pressure (mm Hg)	130.7 (23.7)	234
Diastolic blood pressure (mm Hg)	71.3 (13.1)	234

BMI, body mass index.

**Figure 1:**
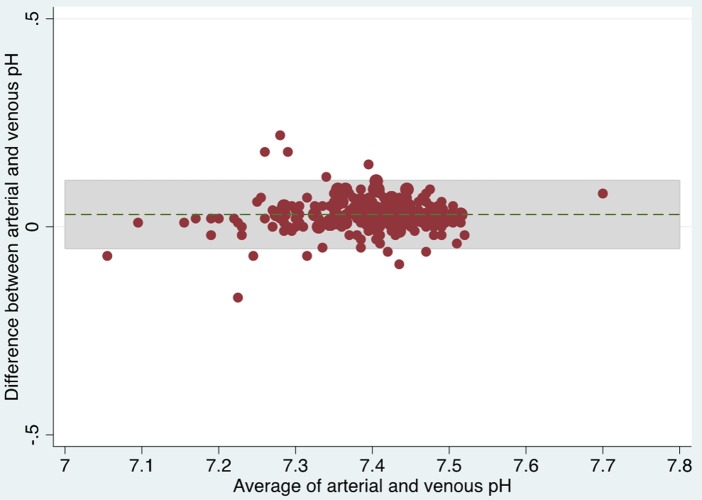
Bland–Altman plot for arterial and venous blood pH levels.

**Figure 2:**
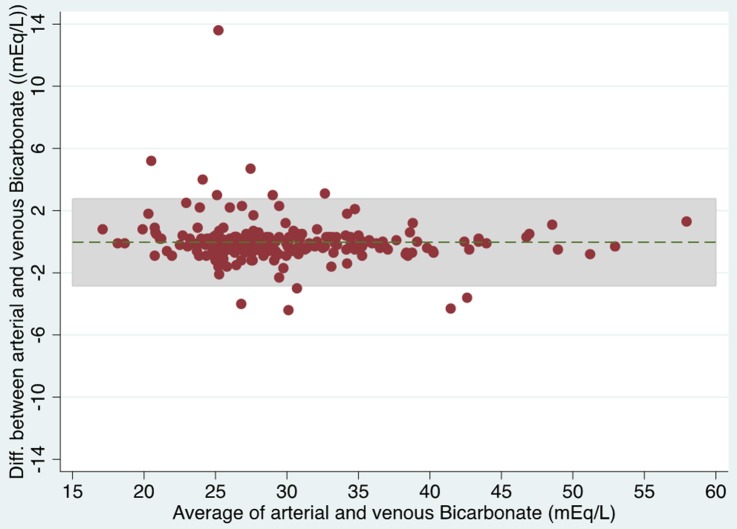
Bland–Altman plot for arterial and venous blood bicarbonate levels.

**Figure 3:**
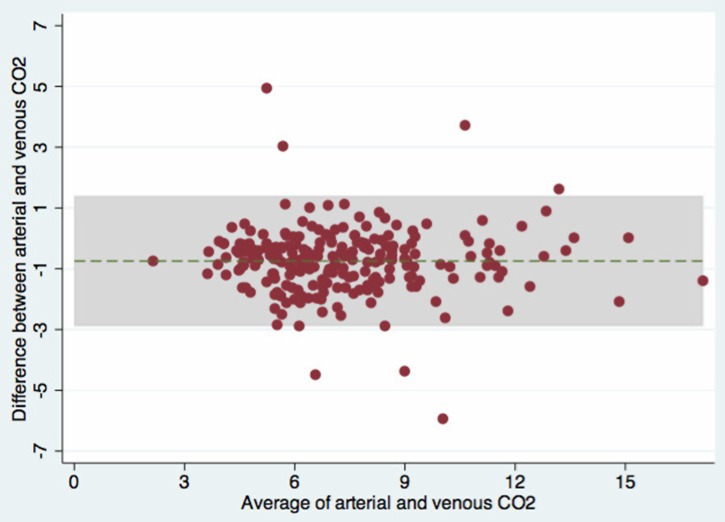
Bland–Altman plot for arterial and venous blood CO_2_ levels.

**Figure 4:**
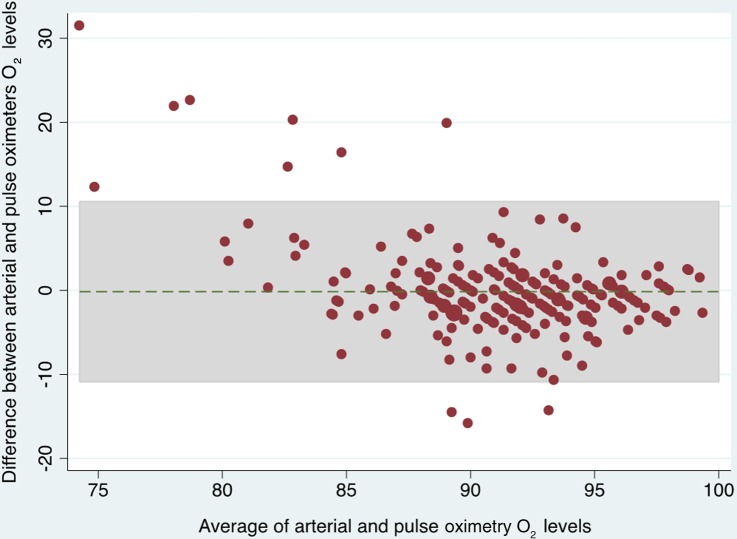
Bland–Altman plot for SaO_2_ and SpO_2_ levels. (This graph only includes patients with a pulse oximetry value of >80%.)

There was good agreement between arterial and venous pH, and between arterial and venous 
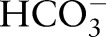
 (figures 1 and 2 and tables 2 and 3), however, the CO_2_ values varied significantly (figure 3). There was also a good agreement between SaO_2_ and SpO_2_ in those patients with an SpO_2_ of ≥80% (figure 4 and tables 2 and 3). These relationships were not significantly different in the 20 patients with an admission systolic blood pressure of <100 mm Hg (see online supplementary material).

**Table 2:** Agreement between arterial and venous pCO_2_, pH and HCO_3_^−^

	ABG (mean) (SD)	VBG (mean) (SD)	Mean difference (ABG–VBG) (95% CI)	95% limits of agreement	N
pH	7.40 (0.09)	7.37 (0.08)	0.03 (0.02 to 0.04)	−0.05 to 0.11	234
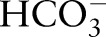 (mEq/L)	29.7 (6.3)	29.7 (6.4)	−0.04 (−0.22 to 0.15)	−2.90 to 2.82	232
pCO_2_ (kPa)	6.89 (2.40)	7.63 (2.41)	−0.75 (CI −0.89 to −0.61)	−2.91 to 1.41	225

ABG, arterial blood gas; VBG, venous blood gas.

**Table 3:** Agreement between SaO_2_ and SpO_2_

	SaO_2_ (mean) (SD)	SpO_2_ (mean) (SD)	Mean difference (SaO_2_–SpO_2_) (95% CI)	95% limits of agreement	N
Oxygen percentage saturation*	91.2 (6.0)	91.0 (4.0)	−0.17 (CI −0.89 to 0.56)	−11.12 to 10.78	224

*In patients with SpO_2_ ≥80%.

### Venous blood cut points for managing acute exacerbations of COPD

Given the relationships observed, we calculated the sensitivity and specificity of a VBG pH and 
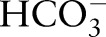
 to correctly identify an arterial pH of ≥7.35, and an arterial 
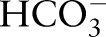
 of ≥21, as well as an SpO_2_ to identify an SaO_2_ of ≥92% (table 4). A venous pH of 7.34, a venous 
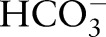
 of 21.45 and an SpO_2_ of 91.5 would have correctly classified 87% (95% CI 82% to 91%), 97% (95% CI 93% to 98%) and 71% (95% CI 65% to 77%) of patients, respectively. In terms of specificity, 96% of patients with an ABG pH of ≥7.35 also had a VBG pH of ≥7.35 (tables 5 and 6).

**Table 4:** Pain score and number of venesection attempts

	n	N
Pain score (median and IQR)		
Arterial pain score	4 (2–5)	187
Venous pain score	1 (0–2)	205
Number of attempts (N and %)		
Arterial number of attempts		
1	162 (69.2)	
2	55 (23.5)	
3	10 (4.3)	
≥4	7 (3.0)	234
Venous number of attempts		
1	211 (90.2)	
2	18 (7.7)	
≥3	5 (2.1)	234

**Table 5:** Predictive performance of venous blood gas parameters

	Venous blood cut-off	AUC	Sensitivity %	Specificity %	Correctly classified %*	N
Arterial pH ≥7.35	7.34	0.92	88.9	95.6	87	234
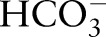 (mEq/L) ≥21	21.45	0.98	96	100	97	232

*Correctly classified refers to percentage of patients correctly classified both above and below given parameter. AUC; area under curve.

**Table 6:** Predictive performance of pulse oximetry

	SpO_2_ cut-off	AUC	Sensitivity %	Specificity %	Correctly classified*	N
SaO_2_	91.5%	0.75	78	72	71	233

*Correctly classified refers to percentage of patients correctly classified both above and below given parameter.

We used current oxygen guidelines^15^ to calculate how many ABG samples may have been avoided if the venous cut points were used instead. Of our 234 patients, 72 (31%) had a venous pH <7.35. Of the 162 with a venous pH ≥7.35, only two had an arterial pH of <7.35.

**Figure 5:**
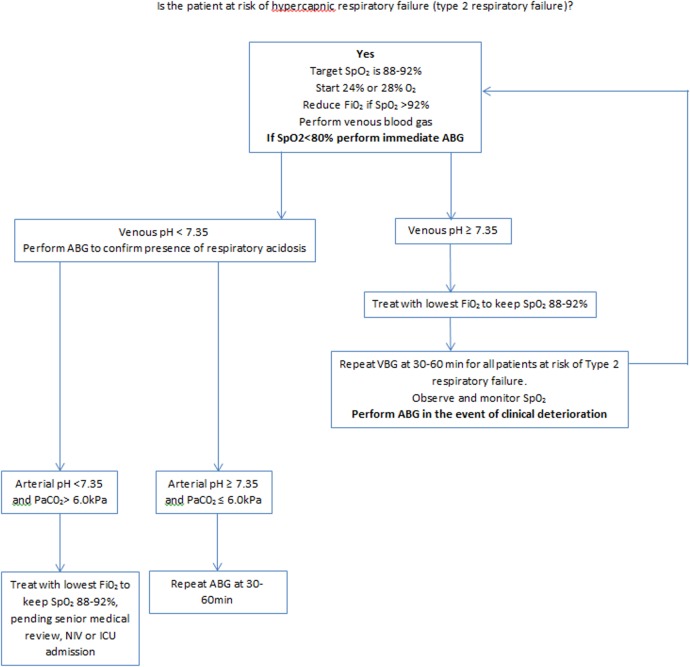
Suggested algorithm for blood gas analysis during COPD exacerbation (adapted from Kelly^10^). ABG, arterial blood gas; ICU, intensive care unit; NIV, non-invasive ventilation.

Consequently, we estimate approximately two-thirds of ABGs can safely be avoided in the initial assessment of COPD exacerbations. This figure does not factor in the repeat attempts needed to obtain arterial blood. Using these data, we suggest a new algorithm for the management of COPD exacerbations based on the current guidelines (figure 5).

### Timing of samples

We assessed the mean difference in time between the paired arterial and venous samples to see if a delay between samples had an effect on the relationship between ABG and VBG parameters. The mean time difference was −4.18 min, SD 16.92, range −58.98 to 78.64 min (arterial–venous). Given the range, we repeated our analysis using the 168 paired samples that were performed within 15 min of each other. This did not affect the relationships (see online supplementary table).

### Pain score

The median pain score was significantly higher for ABG sampling as compared with VBG (p<0.001). In addition, there was a significantly greater number of attempts taken to obtain an ABG sample (69.2% achieved at first attempt) compared with VBG, where 90.2% were obtained at the first attempt (p<0.001) (table 4).

## Discussion

Exacerbations of COPD are a major cause of morbidity and mortality worldwide,^16^ and our local figures reflect this. In 2010, there were 1343 admissions into Nottingham University Hospitals Trust. The management of COPD exacerbations depends upon quickly identifying acute hypercapnic respiratory failure. In this study, we set out to establish if ABG analysis obtained for the initial assessment of COPD exacerbations could be replaced by VBG analysis and pulse oximetry when assessing for acute hypercapnic respiratory failure.

We examined the agreement between ABG and VBG parameters and between ABG and pulse oximetry measurements of oxygen saturation in COPD exacerbations and found acceptable agreement for pH, 
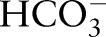
 and for SaO_2_ at an SpO_2_ >80%. We found that 96% of patients with an ABG pH of <7.35 also had a VBG pH of <7.35 and that only two patients were misclassified as having a normal venous pH but a low arterial pH.

A meta-analysis of five studies examining the utility of peripheral VBG analyses in exacerbations of COPD in the emergency department found that there was agreement between arterial and venous pH and 
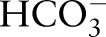
.^9^ The weighted average difference for pCO_2_ was 0.79 kPa (n=440), whereas those for pH and 
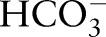
 were 0.028 and 1.34 mmol (n=239), respectively. The relatively weaker relationship seen between SpO_2_ and SaO_2_ at lower levels is unsurprising as commercial pulse oximeters are more accurate at higher oxygen saturations and significantly less accurate below 80%.^17^ ^18^ The proposed algorithm reflects this; any patient with an SpO_2_ of <80% needs an immediate ABG.

Our study has limitations. There was a small time gap between sample acquisition and processing, although results did not change significantly when this was factored in. We also had a pragmatic approach to sample collection which depended upon our junior doctors and specialist nurses; consequently, it was difficult to fully exclude mixed arterial/venous stabs which may explain why the sensitivities and specificities to predict an arterial pH of <7.35 were not 100%. We stress in our algorithm that if there is a risk or actual clinical deterioration an arterial analysis should be performed.

Arterial sampling was more painful than venous and required more attempts. While the pain of arterial sampling can be reduced by using local anaesthetic, it is not widely used.^7^ Although capillary sampling is used in speciality wards, widespread adoption is difficult because of the extra training, resources and time needed. As patients with exacerbations of COPD almost always have venipuncture to obtain samples for full blood count and blood chemistry analysis, VBG analysis can be performed on the same sample.

Our results suggest that the close relationship between venous and arterial acid base parameters, and between oxygen saturations obtained from pulse oximetry and arterial blood, could allow the initial assessment of acute COPD exacerbations to be based on a combined measurement of a VBG pH and SpO_2_. This would mean a change in practice as the current oxygen guidelines published by the British Thoracic Society^15^ state that any patient requiring supplemental oxygen to achieve a target SpO_2_ of 92%–94% should have an arterial blood or arteriolised capillary blood gas performed.

We suggest that arterial sampling is reserved for patients with a venous pH of <7.35. The approach of using venous blood first has obvious benefits. Only one blood draw would be required resulting in less pain and a lower risk of bruising and associated side effects. Less training would be required to initially assess acid/base status, and fewer attempts to draw blood would be needed, needing less equipment and simplifying the care pathway for COPD exacerbations. We conservatively estimate that >66% of ABG attempts would be avoided and replaced by VBG sampling.

## Conclusion

There is a good agreement between pH and 
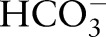
 values derived from venous and arterial blood, and between pulse oximetry and ABG oxygen saturations. These agreements could allow the initial assessment of COPD exacerbations to be based on VBG analysis and pulse oximetry rather than ABG analysis, simplifying the care pathway.

## Supplementary Material

Web supplement

## References

[1] APPG. All party parliamentary group on respiratory health: report on enquiry into respiratory deaths. 2014.

[2] Aubier M, Murciano D, Milic-Emili J, et al Effects of the administration of O_2_ on ventilation and blood gases in patients with chronic obstructive pulmonary disease during acute respiratory failure. Am Rev Respir Dis 1980;122:747–54. 10.1164/arrd.1980.122.5.747 6778278

[3] Hudson LD Survival data in patients with acute and chronic lung disease requiring mechanical ventilation. Am Rev Respir Dis 1989;140 (Pt 2):S19–24. 10.1164/ajrccm/140.2_Pt_2.S19 2669584

[4] Jeffrey AA, Warren PM, Flenley DC Acute hypercapnic respiratory failure in patients with chronic obstructive lung disease: risk factors and use of guidelines for management. Thorax 1992;47:34–40. 10.1136/thx.47.1.34 1539142 PMC463551

[5] NICE. Chronic obstructive pulmonary disease: management of chronic obstructive pulmonary disease in adults in primary and secondary care. London, UK: Royal College of Physicians, 2010. 22319804

[6] Dar K, Williams T, Aitken R, et al Arterial versus capillary sampling for analysing blood gas pressures. BMJ 1995;310:24–5. 10.1136/bmj.310.6971.24 7827548 PMC2548437

[7] Mangera Z, Gunasekera C, Kinley J, et al P113 the use of local anaesthesia in improving the patient experience of arterial blood gases: students and trainers are still not getting the message. Thorax 2014;69 (Suppl 2):A127 10.1136/thoraxjnl-2014-206260.254

[8] Kelly AM, Kyle E, McAlpine R Venous pCO(2) and pH can be used to screen for significant hypercarbia in emergency patients with acute respiratory disease. J Emerg Med 2002;22:15–9. 10.1016/S0736-4679(01)00431-0 11809551

[9] Lim BL, Kelly AM A meta-analysis on the utility of peripheral venous blood gas analyses in exacerbations of chronic obstructive pulmonary disease in the emergency department. Eur J Emerg Med 2010;17:246–8. 10.1097/MEJ.0b013e328335622a 19996974

[10] Kelly AM Review article: can venous blood gas analysis replace arterial in emergency medical care. Emerg Med Australas 2010;22:493–8. 10.1111/j.1742-6723.2010.01344.x 21143397

[11] Kelly AM, Klim S Agreement between arterial and transcutaneous PCO_2_ in patients undergoing non-invasive ventilation. Respir Med 2011;105:226–9. 10.1016/j.rmed.2010.11.010 21131188

[12] Kelly AM, McAlpine R, Kyle E Venous pH can safely replace arterial pH in the initial evaluation of patients in the emergency department. Emerg Med J 2001;18:340–2. 10.1136/emj.18.5.340 11559602 PMC1725689

[13] Kelly AM Can VBG analysis replace ABG analysis in emergency care? Emerg Med J 2014. 10.1136/emermed-2014-204326 25552544

[14] Bland JM, Altman DG Statistical methods for assessing agreement between two methods of clinical measurement. Lancet 1986;327:307–10. 10.1016/S0140-6736(86)90837-8 2868172

[15] O'Driscoll BR, Howard LS, Davison AG BTS guideline for emergency oxygen use in adult patients. Thorax 2008;63 (Suppl 6):vi1–68. 10.1136/thx.2008.102947 18838559

[16] Rabe KF, Hurd S, Anzueto A, et al Global strategy for the diagnosis, management, and prevention of chronic obstructive pulmonary disease: GOLD executive summary. Am J Respir Crit Care Med 2007;176:532–55. 10.1164/rccm.200703-456SO 17507545

[17] Jubran A Pulse oximetry. Crit Care 1999;3:R11–7. 10.1186/cc341 11094477 PMC137227

[18] Wilson BJ, Cowan HJ, Lord JA, et al The accuracy of pulse oximetry in emergency department patients with severe sepsis and septic shock: a retrospective cohort study. BMC Emerg Med 2010;10:9 10.1186/1471-227X-10-9 20444248 PMC2876142

