# Supplementary material for: Using venous blood gas analysis in the assessment of COPD exacerbations: a prospective cohort study
Source: Thorax. 2015 Dec 1;71(3):210–5. doi: 10.1136/thoraxjnl-2015-207573 (PMC4789825; doi:10.1136/thoraxjnl-2015-207573)
Supplement: Web supplement [file thoraxjnl-2015-207573-s1.pdf]

**Online supplement**

**Methods**

Recruitment

Recruitment for this study was based on a convenience approach during routine working hours. Two research nurses were on the wards for a year and approached any patient who met the criteria. The first patient was included on 03/02/2013 and the last patient on 10/01/2014. Patients were flagged by junior doctors and ward nurses if they were eligible. At busy times not all patients were approached and during holiday periods and weekends the numbers approached dropped. Only 12 of the approached patients declined to take part. There were no dropouts as the study only involved one set of paired samples.

**Results**

Agreement between arterial and venous pCO2, pH and HCO3- with samples limited to ones taken within 15 minutes of each other

| | ABG (Mean) (SD) | VBG (Mean) (SD) | Mean Difference (ABG- VBG) (95% CI) | 95% Limits of agreement | N |
|---|---|---|---|---|---|
| pH | 7.41 (0.08) | 7.38 (0.08) | 0.033 (0.027 to 0.038) | -0.04 to 0.11 | 168 |
| $HCO_3^-$ (mEq/L) | 29.38 (6.17) | 29.49 (6.23) | -0.13 (-0.287 to 0.021) | -2.15 to 1.88 | 167 |
| $pCO_2$ (kPa) | 6.66 (2.35) | 7.49 (2.40) | 1.86 (1.45 to 2.26) | -3.26 to 6.96 | 168 |

Agreement between $SaO_2$ and $SpO_2$ with samples limited to ones taken within 15 minutes of each other

| | $SaO_2$ | $SpO_2$ | Mean Difference ($SaO_2$- $SpO_2$) (95% CI) | 95% Limits of agreement | N |
|---|---|---|---|---|---|
| Oxygen percentage saturation* | 90.98 (5.88) | 91.0 (3.97) | 0.04 (-0.85 to 0.93) | -11.43 to 11.5 | 163 |

* in patients with SpO2 >80%

Agreement between arterial and venous pCO2, pH and HCO3- with samples from patients with an initial systolic blood pressure <100mmhg.

| | ABG (Mean) (SD) | VBG ( Mean) (SD) | Mean Difference (ABG- VBG) (95% CI) | 95% Limits of agreement | N |
|---|---|---|---|---|---|
| pH | 7.40 (0.08) | 7.37 (0.07) | 0.02 (-0.004 to 0.05) | -0.54 to 0.11 | 20 |
| $HCO_3^-$ (mEq/L) | 28.1 (4.9) | 28.1 (4.9) | -0.24 (-0.82 to 035) | -2.90 to 2.82 | 20 |
| $pCO_2$ (kPa) | 6.64 (2.07) | 7.24 (1.76) | 1.74 (0.39 to 3.1) | -3.88 to 7.36 | 20 |

Agreement between SaO2 and SpO2 with samples from patients with an initial systolic blood pressure <100mmhg.

| | $SaO_2$ | $SpO_2$ | Mean Difference (SaO2- SpO2) (95% CI) | 95% Limits of agreement | N |
|---|---|---|---|---|---|
| Oxygen percentage saturation* | 90.6 (6.0) | 91.3 (7.3) | 0.62 (CI -2.4to 3.6) | -11.8 to 13.0 | 19 |

* in patients with SpO2 >80%
